# Pulmonary benign metastasizing leiomyoma presenting as advanced lung cancer: A case report

**DOI:** 10.1016/j.rmcr.2026.102500

**Published:** 2026-09-28

**Authors:** Jeffrey Medina-Medina, Luis Vargas-González, Katia Munguia-Galeano, Norwing García-García, Melvin Romero-Jarquin, Christian Toval-Ruiz, Manuel López-Urbina

**Affiliations:** a Department of Internal Medicine, Hospital Dr. Mauricio Abdalah, Chinandega, Nicaragua; b Department of Pathology, Hospital Dr. Mauricio Abdalah, Chinandega, Nicaragua; c Master's Degree Program in Epidemiology, Universidad Tecnológica La Salle, León, Nicaragua

**Keywords:** Pulmonary benign metastasizing leiomyoma, Uterine leiomyoma, Pulmonary nodules, Lung cancer, Immunohistochemistry, Differential diagnosis

## Abstract

**Background:**

Pulmonary benign metastasizing leiomyoma (PBML) is a rare extrauterine smooth muscle neoplasm that predominantly affects women with a history of uterine leiomyoma. Because its clinical and radiological manifestations frequently resemble advanced pulmonary malignancy, establishing the diagnosis remains challenging. Herein, we describe an unusual presentation characterized by a dominant pulmonary mass and diffuse bilateral pulmonary nodules, initially suggesting advanced lung cancer with intrapulmonary metastases.

**Case presentation:**

We report the case of a 38-year-old woman with a previous history of total hysterectomy for symptomatic uterine leiomyomatosis who presented with progressive exertional dyspnea and persistent dry cough. Chest imaging revealed a dominant left lower lobe pulmonary mass associated with numerous bilateral pulmonary nodules, initially raising strong suspicion of advanced lung cancer with intrapulmonary metastases. Extensive microbiological and oncological investigations were unrevealing. Surgical lung biopsy demonstrated benign spindle-cell proliferation without atypia or mitotic activity, while immunohistochemistry confirmed pulmonary benign metastasizing leiomyoma. ^18F-FDG PET/CT excluded hypermetabolic extrapulmonary disease. The patient was managed conservatively with clinical and radiological surveillance and remained clinically stable with symptomatic improvement.

**Conclusion:**

PBML should be considered in the differential diagnosis of women with a history of uterine leiomyoma who present with multiple pulmonary nodules or a dominant pulmonary mass. Histopathological and immunohistochemical confirmation is essential to establish the diagnosis and avoid unnecessary oncologic treatment. Conservative surveillance may represent an appropriate management strategy in selected patients with indolent disease. Given the short follow-up interval in this report, longer prospective surveillance is needed to confirm the durability of this approach.

## 1 Background

Pulmonary benign metastasizing leiomyoma (PBML) is a rare smooth muscle neoplasm defined by histologically benign uterine leiomyoma occurring at distant extrauterine sites, most commonly the lungs, typically after hysterectomy or myomectomy [1,2].

The diagnosis of PBML is challenging because its clinical and radiological presentation frequently mimics primary lung cancer or metastatic disease, and misdiagnosis can lead to unnecessary invasive procedures and inappropriate oncologic therapy. Here, we report a case of PBML presenting as a dominant pulmonary mass with diffuse bilateral pulmonary nodules, initially raising strong suspicion of advanced lung cancer with intrapulmonary metastases [2,3]. This case report was prepared in accordance with the CARE (CAse REport) guidelines [4].

## 2 Case Presentation

### 2.1 Clinical presentation

A 38-year-old Nicaraguan woman presented to our hospital in February 2026 because of a two-week history of persistent dry cough and progressive exertional dyspnea. She denied fever, hemoptysis, chest pain, weight loss, or night sweats. Empirical symptomatic treatment for a presumed upper respiratory tract infection had failed to relieve her symptoms, prompting further diagnostic investigation.

Her medical history was significant for symptomatic uterine leiomyomatosis, for which she had undergone a total hysterectomy with ovarian preservation in January 2022. She had remained asymptomatic until the onset of respiratory symptoms approximately four years later. She had no history of smoking, alcohol consumption, hormone replacement therapy, chronic medical conditions or family history of malignancy or hereditary disease. From a psychosocial standpoint, she worked as a cashier at a small café, and reported a supportive environment without significant occupational or psychosocial stressors.

On admission, the patient was hemodynamically stable and in no apparent distress at rest despite reporting progressive exertional dyspnea. Her body mass index was 29.3 kg/m^2^, and oxygen saturation while breathing ambient air was 98%. Pulmonary auscultation revealed bilateral inspiratory crackles, whereas the remainder of the physical examination was unremarkable. The overall chronological sequence of the patient's clinical course and diagnostic evaluation is summarized in Fig. 1.

**Fig. 1:**
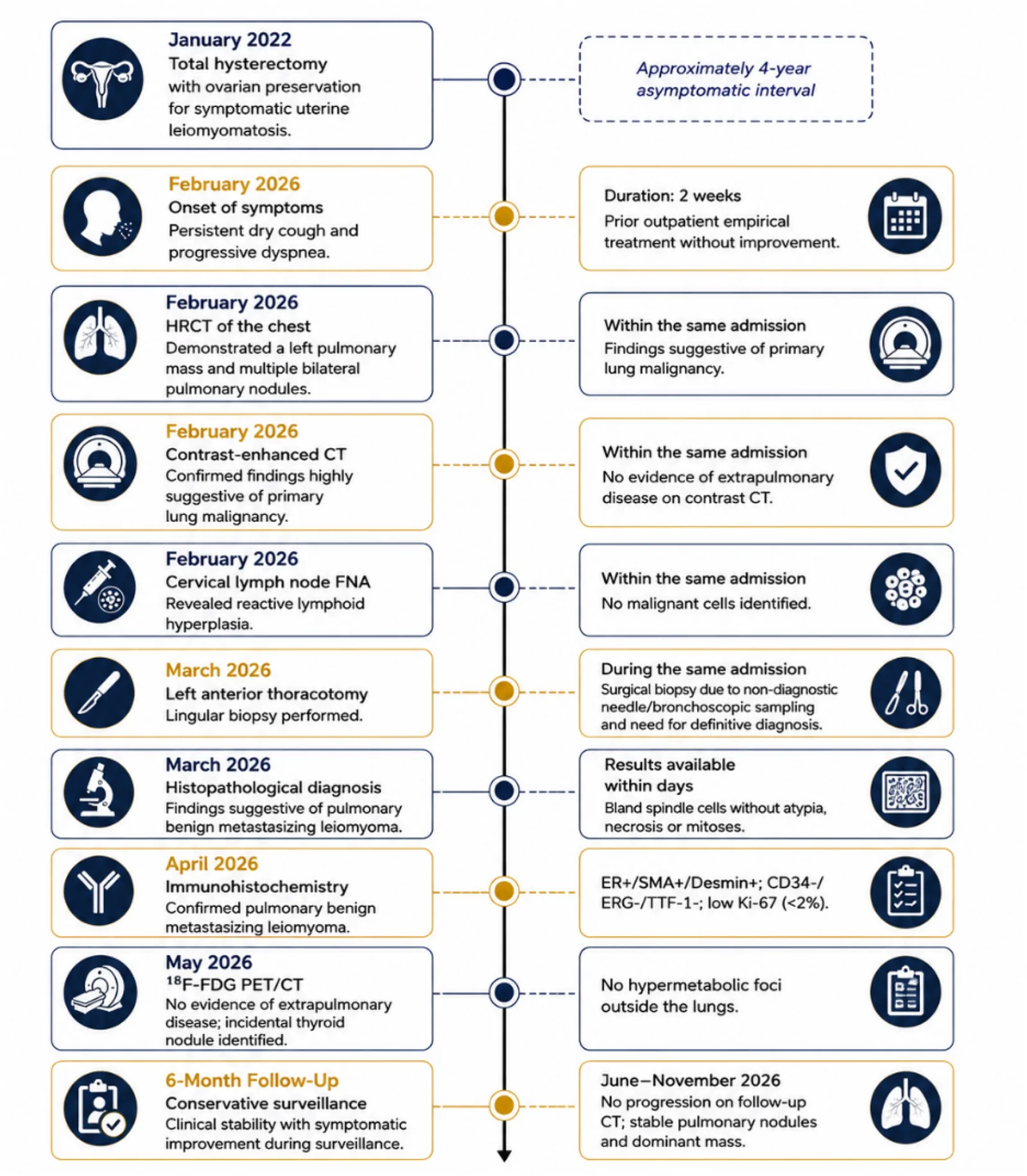
Chronological summary of the diagnostic work-up and management of pulmonary benign metastasizing leiomyoma, from gynecologic surgery to histopathological confirmation and clinical follow-up. Abbreviations: CT, computed tomography; FNA, fine-needle aspiration; HRCT, high-resolution computed tomography; PET/CT, positron emission tomography/computed tomography; FDG, fluorodeoxyglucose; PBML, pulmonary benign metastasizing leiomyoma. The chronological sequence summarizes the patient's clinical course, beginning with total hysterectomy for symptomatic uterine leiomyomatosis in January 2022, followed by an approximately 4-year asymptomatic interval before the onset of respiratory symptoms. The timeline illustrates the sequential diagnostic evaluation, including chest HRCT, contrast-enhanced CT, cervical lymph node fine-needle aspiration, surgical lung biopsy, histopathological and immunohistochemical confirmation of pulmonary benign metastasizing leiomyoma, ^18F-FDG PET/CT, and subsequent conservative clinical and radiological surveillance.

### 2.2 Initial laboratory evaluation

Initial laboratory investigations were essentially unremarkable. The complete blood count showed no leukocytosis, while inflammatory biomarkers, including C-reactive protein and procalcitonin, were within normal limits. Blood cultures remained sterile, HIV serology was nonreactive, and microbiological investigations for pulmonary tuberculosis, including sputum acid-fast bacilli smear microscopy and the Xpert MTB/RIF assay, were negative. Collectively, these findings made acute infectious etiologies, including bacterial pneumonia, septic pulmonary emboli, and pulmonary tuberculosis, considerably less likely, prompting further investigation of noninfectious causes of diffuse pulmonary nodules.

### 2.3 Radiological assessment

Given the persistence of respiratory symptoms despite an unrevealing initial laboratory evaluation, high-resolution computed tomography (HRCT) of the chest was performed. The examination revealed a large left lower lobe (parahilar) pulmonary lesion measuring 73 × 48 mm, together with numerous well-circumscribed bilateral pulmonary nodules measuring up to 42 × 35 mm, randomly distributed throughout both lungs. No pleural effusion or mediastinal lymphadenopathy was identified. These findings raised a strong suspicion of advanced lung cancer with diffuse intrapulmonary metastases and prompted further diagnostic evaluation.

To further characterize the pulmonary lesions and investigate a possible primary malignancy or extrapulmonary disease, contrast-enhanced computed tomography of the brain, chest, abdomen, and pelvis was performed. The study confirmed a heterogeneous left lower lobe pulmonary mass measuring 77 × 50 × 37 mm, accompanied by multiple bilateral pulmonary nodules. No intracranial lesions, mediastinal or retroperitoneal lymphadenopathy, or abdominal metastatic disease were identified. Incidental findings included hepatomegaly, cholelithiasis, mild gastric wall thickening, and a simple right ovarian cyst. Collectively, these findings further strengthened the suspicion of advanced lung cancer with diffuse pulmonary metastases. Representative HRCT and contrast-enhanced CT findings are shown in Fig. 2.

**Fig. 2:**
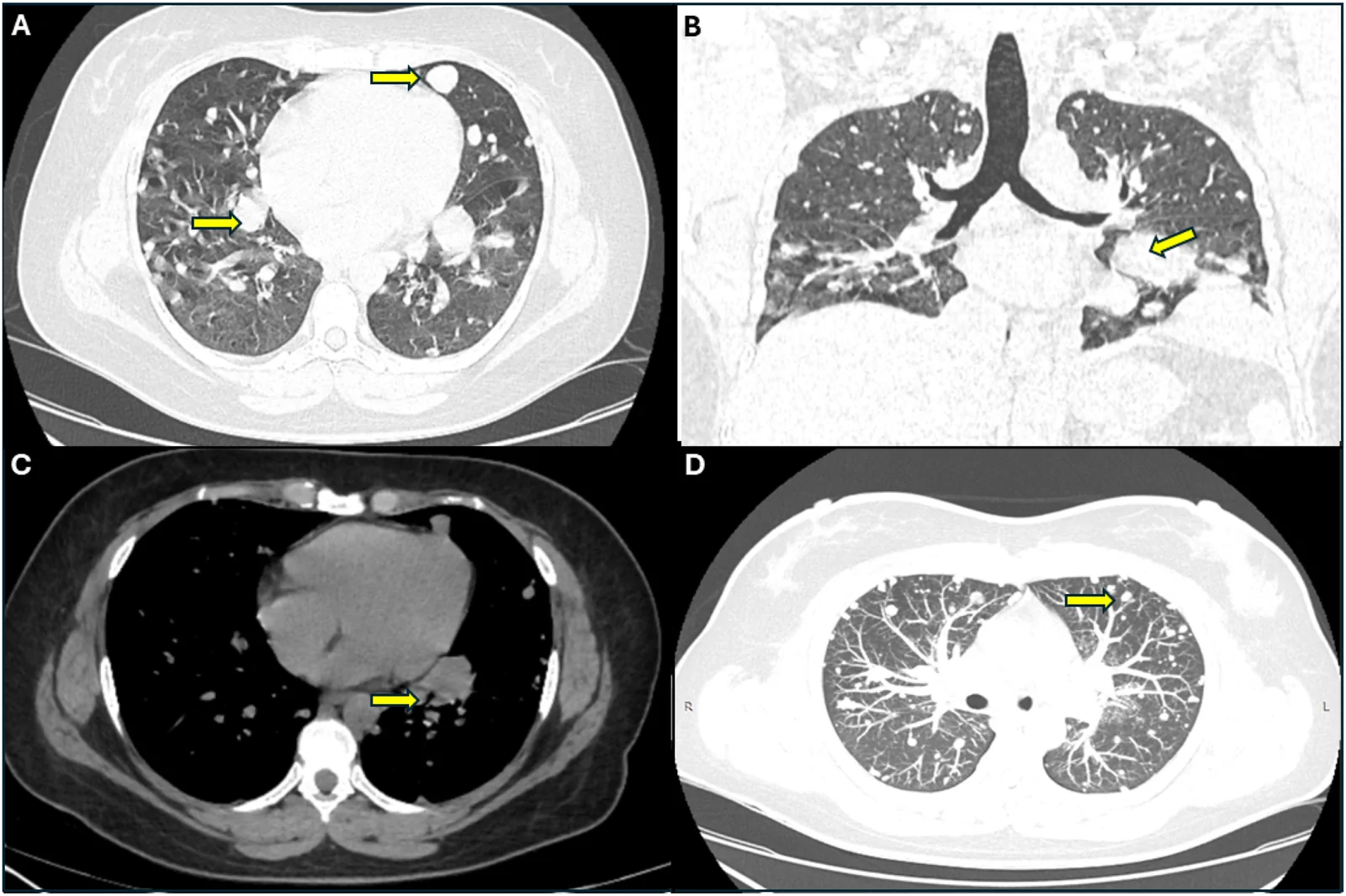
Chest computed tomography findings demonstrating a dominant pulmonary mass and multiple bilateral pulmonary nodules (A) Axial chest computed tomography image demonstrating multiple bilateral well-circumscribed pulmonary nodules (arrows). (B) Coronal reconstruction showing a dominant left lower lobe pulmonary lesion associated with additional bilateral nodules (arrow). (C) Axial mediastinal window depicting a left parahilar soft tissue lesion adjacent to vascular structures without evidence of invasion (arrow). (D) Maximum intensity projection (MIP) reconstruction highlighting the diffuse hematogenous distribution of multiple pulmonary nodules (arrow).

### 2.4 Diagnostic work-up

Ultrasound-guided fine-needle aspiration of a cervical lymph node demonstrated reactive lymphoid hyperplasia without evidence of malignancy.

Upper gastrointestinal endoscopy excluded a synchronous gastrointestinal malignancy.

### 2.5 Surgical biopsy

Following multidisciplinary discussion, surgical biopsy was considered necessary to obtain an adequate tissue sample for definitive diagnosis. Under general anesthesia, the patient underwent a left anterior thoracotomy. Intraoperatively, multiple violaceous nodules measuring approximately 4–6 mm were identified within the lingula without involvement of the visceral pleura or associated pleural effusion. A wedge resection of the lingula was performed using a surgical stapling device to obtain representative tissue for histopathological examination. A 19-Fr thoracic drain was placed at the end of the procedure, which was completed without intraoperative complications.

### 2.6 Histopathological examination

Macroscopic examination of the surgical specimen revealed multiple well-circumscribed intraparenchymal nodules with a soft consistency and well-defined borders, as shown in Fig. 3. The cut surface was predominantly dark brown with focal whitish areas and a fibrous appearance. Microscopic examination demonstrated multiple well-circumscribed nodular proliferations composed of bland spindle cells arranged in intersecting fascicles within a hemorrhagic stroma. Scattered entrapped glandular structures lined by columnar epithelium were identified within some nodules. No cytologic atypia, coagulative necrosis, or mitotic figures were observed. The surrounding pulmonary parenchyma showed focal edema and hemorrhage. Taken together, the histomorphological features were considered compatible with pulmonary benign metastasizing leiomyoma, and immunohistochemical studies were subsequently performed to confirm the diagnosis. Representative hematoxylin and eosin photomicrographs are shown in Fig. 4.

**Fig. 3:**
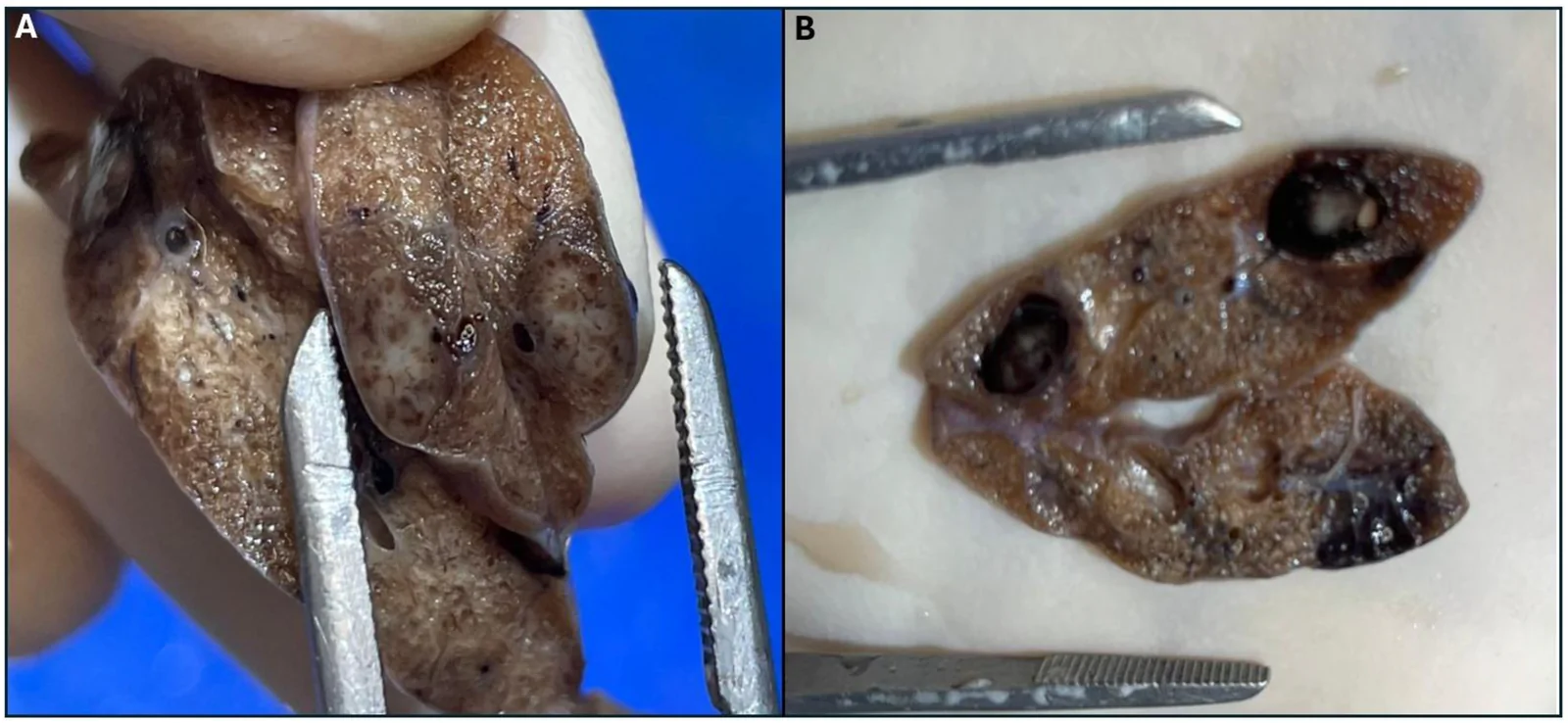
Gross pathological findings of pulmonary nodules (A) Surgical lung specimen demonstrating multiple firm, well-circumscribed whitish nodules involving the pulmonary parenchyma. (B) Sectioned pulmonary specimen showing discrete intraparenchymal nodules with smooth cut surfaces and focal hemorrhagic changes.

**Fig. 4:**
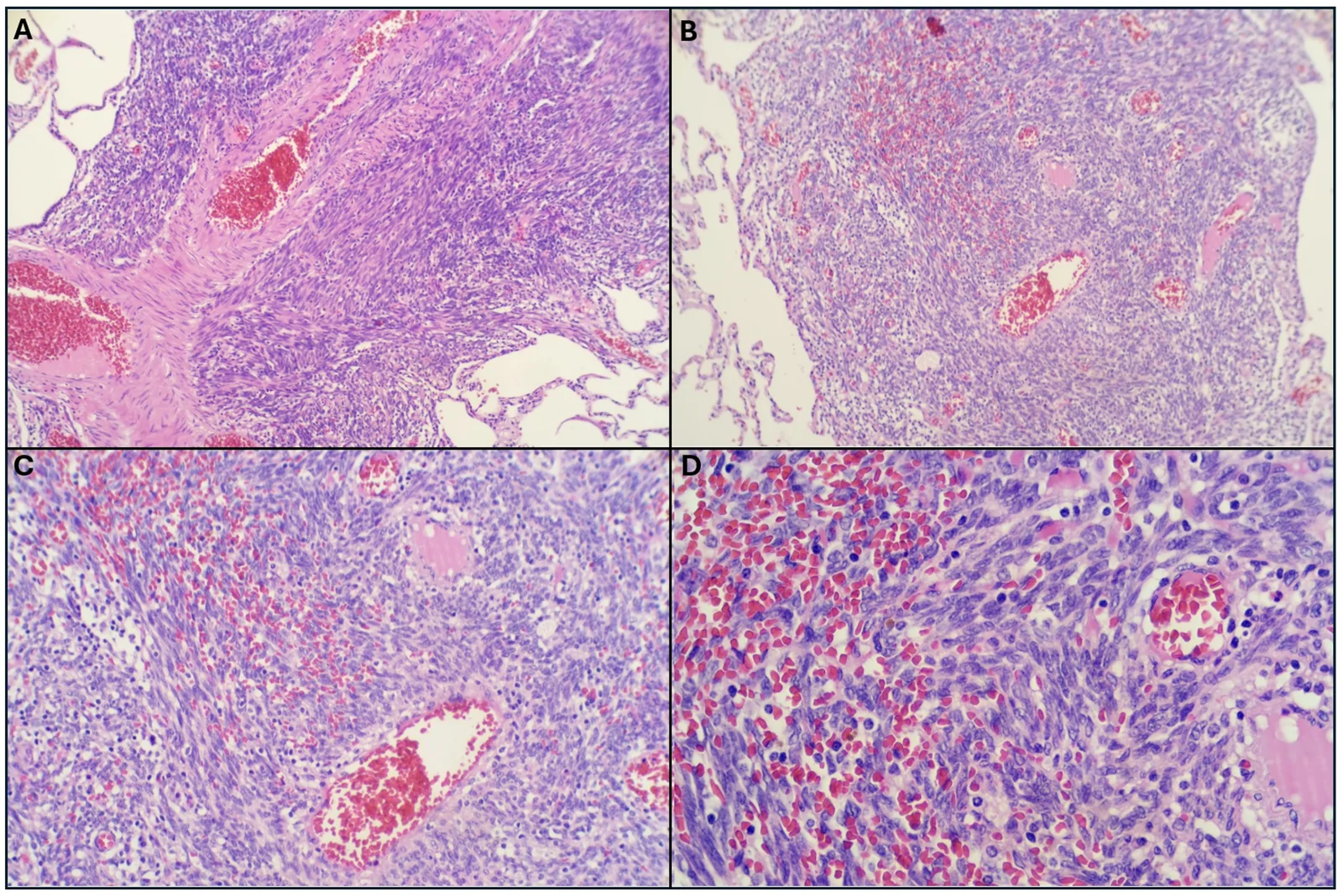
Histopathological findings of pulmonary benign metastasizing leiomyoma (A) Low-power hematoxylin and eosin (H&E) photomicrograph showing spindle cell proliferation arranged in fascicles within the pulmonary parenchyma (H&E, ×40). (B) Intermediate magnification demonstrating intersecting bundles of bland smooth muscle cells without significant atypia or necrosis (H&E, ×100). (C) Higher magnification highlighting the absence of marked pleomorphism or mitotic activity (H&E, ×200). (D) High-power view showing uniform spindle-shaped cells with eosinophilic cytoplasm and elongated nuclei, consistent with benign smooth muscle proliferation (H&E, ×400).

### 2.7 Immunohistochemical characterization

To further characterize the spindle-cell proliferation and confirm smooth muscle differentiation, immunohistochemical staining was performed. The tumor cells showed diffuse positivity for smooth muscle actin (SMA), desmin, and estrogen receptor (ER), whereas CD34, ERG, and thyroid transcription factor-1 (TTF-1) were negative. The Ki-67 proliferation index was approximately 1%, supporting a low proliferative activity. Taken together, these immunophenotypic findings confirmed the diagnosis of pulmonary benign metastasizing leiomyoma. The immunohistochemical profile confirming smooth muscle differentiation is illustrated in Fig. 5, and the full immunohistochemical panel is summarized in Table 2.

**Fig. 5:**
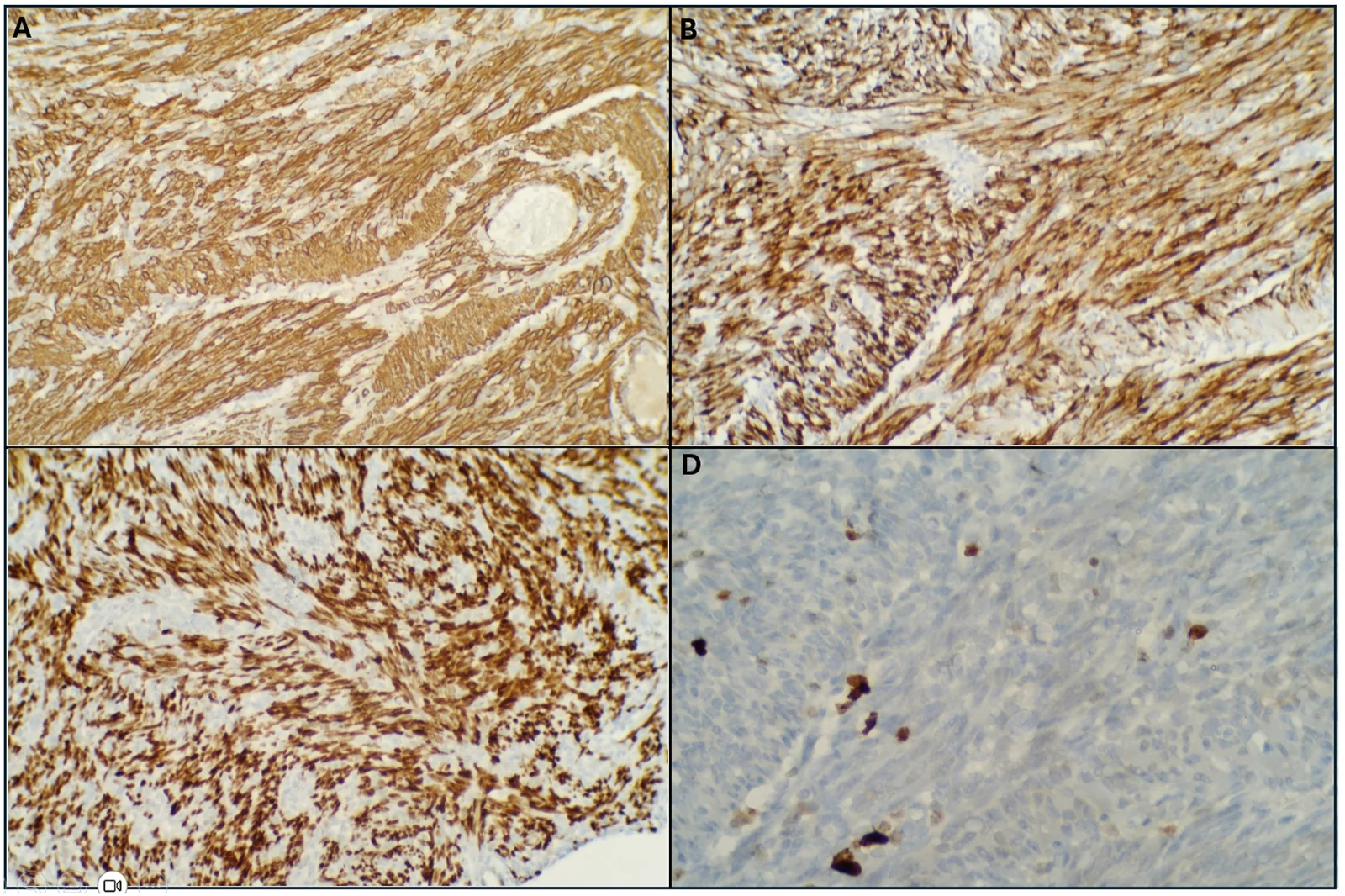
Immunohistochemical characterization of pulmonary benign metastasizing leiomyoma (A) Diffuse cytoplasmic positivity for smooth muscle actin in tumor cells (IHC, ×200). (B) Strong desmin expression confirming smooth muscle differentiation (IHC, ×200). (C) Nuclear positivity for estrogen receptor within tumor cells (IHC, ×200). (D) Low proliferative activity demonstrated by Ki-67 labeling index of approximately 1% (IHC, ×400).

### 2.8 FDG PET/CT findings

^18F-FDG PET/CT demonstrated low metabolic activity confined to the pulmonary lesions without evidence of hypermetabolic extrapulmonary disease, supporting conservative management. An incidental thyroid nodule was managed according to current surveillance recommendations. Representative ^18F-FDG PET/CT images are presented in Fig. 6.

**Fig. 6:**
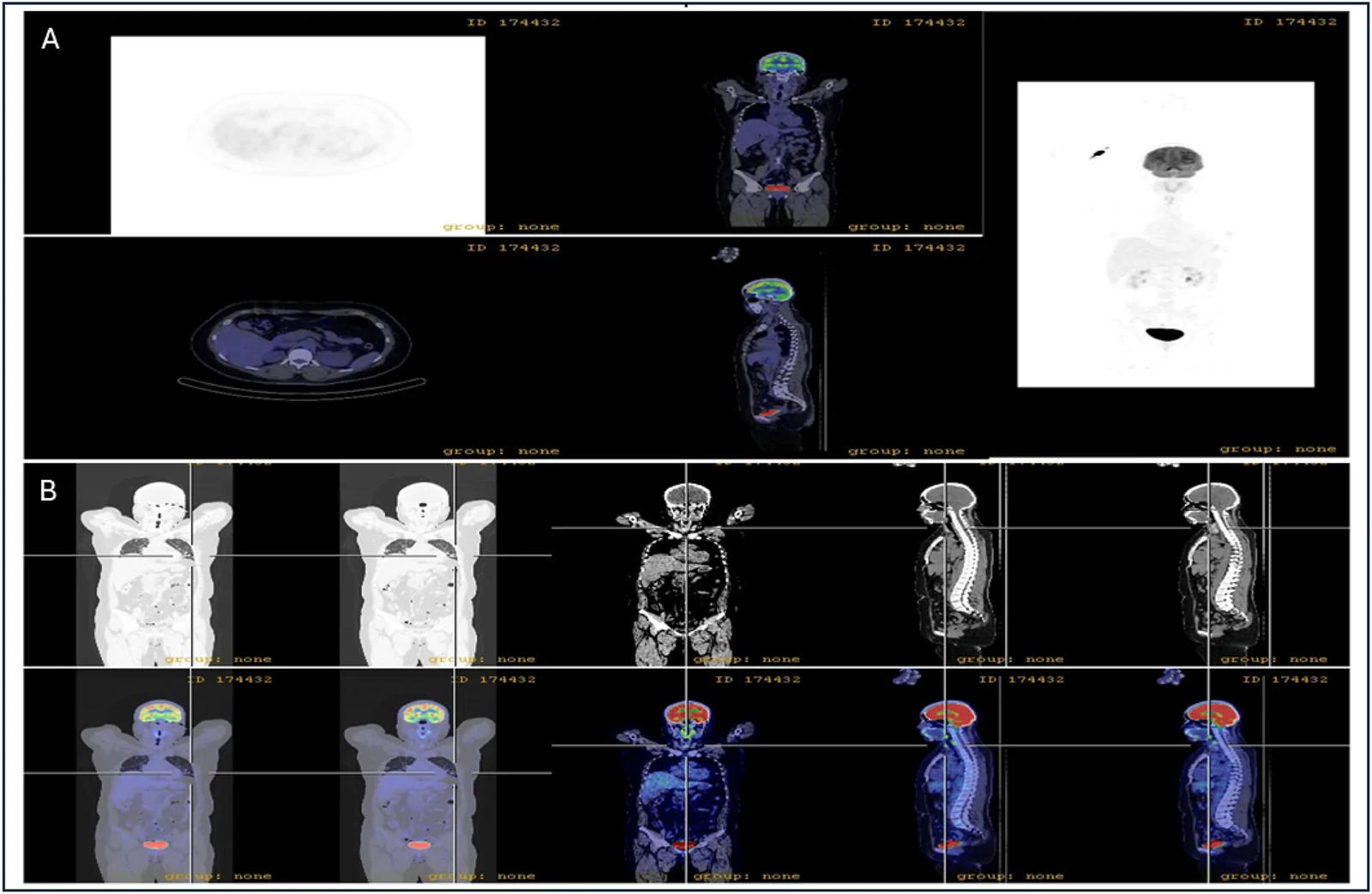
^18F-FDG PET/CT findings in pulmonary benign metastasizing leiomyoma. (A) Whole-body ^18F-FDG PET/CT demonstrating multiple bilateral pulmonary nodules with low metabolic activity and no evidence of hypermetabolic extrapulmonary disease. Axial, coronal, sagittal, and maximum-intensity projection (MIP) images are shown. (B) Fused PET/CT images confirming that the pulmonary lesions exhibit low ^18F-FDG uptake without evidence of mediastinal, abdominal, or skeletal metastatic disease. These findings support disease confined to the lungs and are consistent with the indolent biological behavior of pulmonary benign metastasizing leiomyoma.

### 2.9 Follow-up

Given the patient's preserved functional status, favorable clinical evolution, absence of extrapulmonary disease, and the indolent histopathological characteristics of the tumor, no additional surgical intervention or hormonal therapy was initiated. Instead, she was managed with close clinical and radiological surveillance. At the 6 month follow-up, the patient reported marked improvement and was free of respiratory symptoms and remained clinically stable, with no evidence of disease progression. A follow-up chest radiograph obtained at that time demonstrated stability of the pulmonary nodules and the dominant pulmonary lesion, without evidence of progression.

## 3 Discussion

Pulmonary benign metastasizing leiomyoma (PBML) is a rare benign extrauterine smooth muscle tumor that usually occurs years after hysterectomy for uterine leiomyoma [2,3] with the lungs as the most common metastatic site [5,6], and it frequently mimics metastatic or primary lung cancer despite its benign histology.

The diagnosis of PBML is particularly challenging because its clinical and radiological manifestations frequently overlap with those of lung cancer and metastatic disease [3,7]. Although many patients are diagnosed incidentally during imaging performed for unrelated indications, symptomatic presentations with a dominant pulmonary mass accompanied by numerous bilateral pulmonary nodules are uncommon and may strongly suggest advanced thoracic malignancy [1,2]. In the present case, the combination of progressive exertional dyspnea, a large left lower lobe mass, and diffuse bilateral pulmonary nodules initially raised a high suspicion of advanced lung cancer with intrapulmonary metastases, prompting an extensive multidisciplinary diagnostic evaluation before a definitive diagnosis was established through surgical biopsy and immunohistochemical analysis.

A comparison with previously reported series further contextualizes the present case (Table 1). Fan et al. described 23 patients with a median age at diagnosis of 46 years and a median interval of 10.5 years between gynecologic surgery and PBML diagnosis, with multiple bilateral nodules as the predominant radiologic pattern [3]. Su et al. [8] reported a median interval of 6.0 years among patients aged 45 years or younger. Smaller series, including that of Wojtyś et al., likewise described patients presenting between the fifth and sixth decades of life [2]. In contrast, our patient presented at a younger age (38 years) and after a shorter latency period (4 years), and the coexistence of a dominant pulmonary mass with diffuse bilateral micronodulation represents a less commonly reported combined pattern that more closely mimics primary lung cancer with intrapulmonary metastases.

**Table 1:** Comparison of the present case with previously reported series of pulmonary benign metastasizing leiomyoma (PBML).

Study	n	Age at diagnosis	Interval from gyn. surgery	Predominant pattern	Management	Outcome
Fan et al., 2020 [3]	23	Median 46 y (36–62)	Median 10.5 y (2–15)	Multiple bilateral nodules (78.3%)	Observation; BSO; hormonal therapy	8-y follow-up: CR 22.2%, PR 16.7%, SD 50%, PD 11.1%
Su et al., 2023 [8]	65	≤45 y (by design)	Median 6.0 y	Bilateral solid lesions (60.9%)	Observation (16.7%)	Stable at median 18-mo follow-up
Wojtyś et al., 2022 [2]	3	46–53 y	12 y and 17 y (2/3 cases)	2 multiple-nodule, 1 solitary mass	Individualized	Symptom-free at presentation
Barnas et al., 2017 [6]	Pooled	Mean 47.3 y	Variable, no significant relation	Not pooled quantitatively	Variable	Not pooled quantitatively
Present case	1	38 y	4 y	Dominant mass (73–77 mm) + diffuse bilateral nodules	Conservative surveillance	Clinically stable, asymptomatic improvement

Su et al. [8] included a total cohort of 65 women aged ≤45 years (56 cases from a systematic literature review plus 9 institutional cases); individual variables in the original report were summarized over slightly smaller subgroups with available data (e.g., N = 60 for age, N = 64 for radiographic pattern).

**Table 2:** Immunohistochemical panel and its diagnostic significance.

Marker	Result	Diagnostic significance
SMA	Positive	Confirms smooth muscle differentiation
Desmin	Positive	Confirms myogenic/smooth muscle origin
ER	Positive	Supports uterine (Müllerian) smooth muscle origin
CD34	Negative	Helps exclude a vascular or solitary fibrous tumor
ERG	Negative	Excludes a vascular-derived neoplasm
TTF-1	Negative	Argues against primary lung (pulmonary) carcinoma
Ki-67	∼1%	Confirms a low proliferative activity, consistent with a benign process

A structured diagnostic approach is essential in women presenting with multiple pulmonary nodules and a history of uterine leiomyoma [1,3]. Infectious diseases, particularly pulmonary tuberculosis in endemic regions, lung cancer, metastatic disease, and rare benign pulmonary neoplasms, including PBML, should all be considered in the differential diagnosis [7,9]. In this case, microbiological investigations, cross-sectional imaging, cytological evaluation, endoscopy, and surgical lung biopsy systematically excluded alternative diagnoses before histopathological and immunohistochemical analysis established the diagnosis. The stepwise approach followed is summarized in the author-proposed algorithm in Fig. 7.

**Fig. 7:**
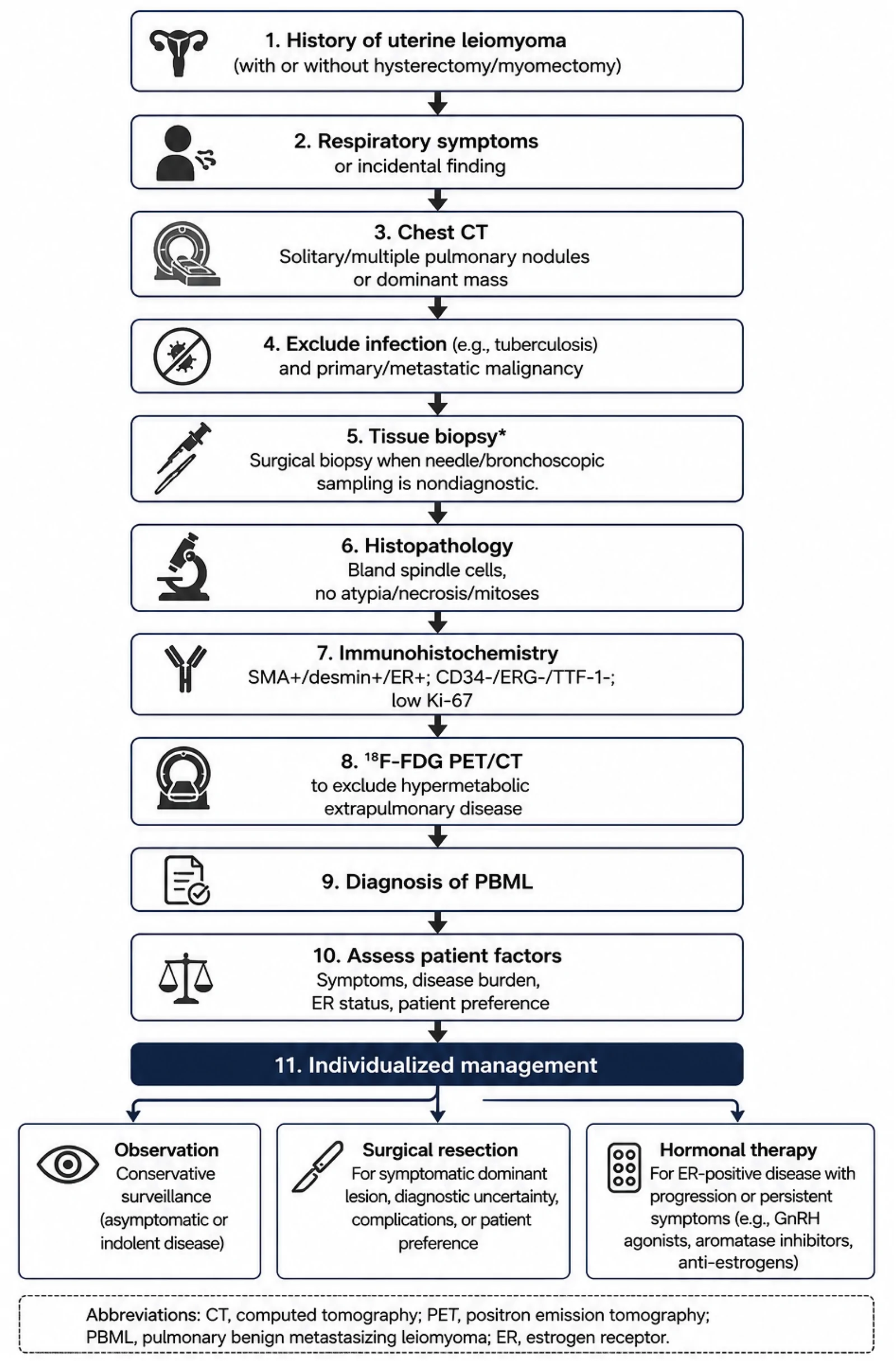
Author-proposed diagnostic algorithm for suspected pulmonary benign metastasizing leiomyoma (PBML), based on the current literature and the diagnostic pathway followed in this case.

Surgical biopsy via anterior thoracotomy and wedge resection was favored over less invasive alternatives, such as flexible bronchoscopy or CT-guided transthoracic needle biopsy, because the lingular nodules were small (4–6 mm) and predominantly subpleural, making transbronchial sampling unlikely to yield adequate tissue and needle biopsy prone to crush artifact and limited material for a comprehensive immunohistochemical panel [10]. An architecturally intact surgical specimen was considered necessary to reliably assess the growth pattern, exclude necrosis or increased mitotic activity, and confidently exclude the histological mimickers discussed below. The surgical approach also permitted complete excision of the sampled nodules.

Histopathological examination remains the diagnostic gold standard for PBML because clinical and radiological findings alone are insufficient to distinguish this entity from primary lung carcinoma, metastatic disease, or other spindle-cell neoplasms of the lung [2,5]. The characteristic histological appearance consists of well-circumscribed fascicles of cytologically bland smooth muscle cells with minimal mitotic activity and absence of necrosis or significant atypia [5,11]. Immunohistochemistry plays a complementary role by confirming smooth muscle differentiation and demonstrating hormone receptor expression, while excluding other spindle-cell tumors through an appropriate immunophenotypic profile [1,5]. In our case, the combined histomorphological and immunohistochemical findings established the diagnosis with confidence and guided subsequent conservative management.

The histopathological differential diagnosis of a pulmonary spindle-cell lesion includes several entities that may closely mimic PBML. Metastatic leiomyosarcoma typically shows cytologic atypia, coagulative necrosis, and an elevated mitotic count and Ki-67 index, none of which were present in our specimen. Perivascular epithelioid cell tumor (PEComa) characteristically displays epithelioid cells with clear-to-granular cytoplasm and coexpression of melanocytic markers (HMB-45, Melan-A) alongside smooth muscle markers, a morphology not observed in our uniformly spindled tumor. Spindle-cell (sarcomatoid) carcinoma generally shows cytokeratin or TTF-1 positivity together with marked cytologic atypia, both excluded by the negative TTF-1 staining and bland cytomorphology in our case. Taken together, the combination of bland spindle-cell morphology, diffuse SMA, desmin, and ER positivity, a low Ki-67 index, and negative CD34, ERG, and TTF-1 allowed confident exclusion of these mimickers and supported the diagnosis of PBML [9].

^18F-FDG PET/CT has an emerging but still limited role in the evaluation of PBML. Because these lesions have low proliferative activity, FDG uptake is characteristically low, which helps exclude a hypermetabolic malignant process but cannot alone confirm a benign diagnosis, since some low-grade sarcomas and indolent metastases can likewise show minimal tracer uptake. In this case, PET/CT was used mainly to exclude occult extrapulmonary disease, and its findings were interpreted alongside — not as a substitute for — histopathological and immunohistochemical confirmation.

Because of its rarity, the optimal management of PBML remains controversial in the absence of standardized treatment guidelines [1,2]. Reported strategies include surgical resection, hormonal suppression via oophorectomy or anti-estrogen therapy, and observation in stable patients [3,6]. Treatment should be individualized according to disease burden, symptom severity, and receptor status [1,2]. In our case, the absence of extrapulmonary disease on ^18F-FDG PET/CT, together with the low proliferative index and progressive clinical improvement, contributed to the decision for conservative surveillance following multidisciplinary discussion. Despite ER positivity, immediate hormonal suppression was deferred given the patient's clinical stability and low-volume indolent disease, and was reserved for consideration should disease progression occur.

Although PBML is generally considered an indolent disease, its initial presentation may closely resemble advanced pulmonary malignancy, particularly when a dominant pulmonary mass coexists with diffuse bilateral nodules, underscoring the importance of a systematic diagnostic approach integrating clinical history, imaging, histopathology, and immunohistochemistry before initiating definitive oncologic treatment [1,3]. This case adds to existing reports suggesting that carefully selected patients with PBML — those with localized pulmonary disease and favorable clinicopathological characteristics such as bland cytomorphology, a low Ki-67 index, and absence of extrapulmonary disease on PET/CT — may be managed conservatively, at least in the short term, rather than with surgery or hormonal therapy [2,6]. Long-term follow-up nonetheless remains essential, because delayed progression has been reported in isolated cases despite the generally indolent biological behavior of PBML.

### 3.1 Limitations

This report has several limitations inherent to the single-case design. As with any individual case report, our findings cannot be generalized, and causal or prognostic inferences are necessarily limited. Follow-up was restricted to 6 months, which is shorter than the multi-year surveillance periods reported in larger series and may not capture late disease progression, a phenomenon occasionally described in PBML; this limitation is particularly relevant given the size of the dominant pulmonary mass in our patient (77 mm), and the 6-month interval reported here should be regarded as an early, not definitive, demonstration of the safety of conservative management — longer prospective surveillance is required before firm conclusions about the durability of this strategy can be drawn. In addition, cytogenetic and clonality studies (e.g., androgen receptor allelic inactivation or telomere length analysis), which have been used in prior series to establish a clonal relationship between pulmonary and uterine lesions, were not performed in our patient, as the diagnosis was established on histomorphological and immunohistochemical grounds alone.

## 4 Conclusion

Pulmonary benign metastasizing leiomyoma should remain an important differential diagnosis in women with a history of uterine leiomyoma who present with multiple pulmonary nodules or a dominant pulmonary mass, even years after hysterectomy or when the interval since gynecological surgery is shorter than classically described. For pulmonologists, internists, radiologists, and pathologists alike, recognition of this rare entity requires a systematic diagnostic approach integrating clinical history, imaging, histopathology, and immunohistochemistry — supported, when indicated, by ^18F-FDG PET/CT to exclude extrapulmonary disease. Early diagnostic confirmation may prevent unnecessary invasive oncologic work-up and treatment and facilitate individualized management, including conservative surveillance with long-term follow-up in carefully selected patients.

### 4.1 Patient perspective

The patient reported that the period of diagnostic uncertainty, during which a malignant process could not be excluded, was the most distressing part of her illness experience. She described substantial relief upon learning that her lungs findings corresponded to a benign process rather than cancer, and she expressed satisfaction with the conservative, surveillance-based approach, which allowed her to avoid additional surgery or hormonal treatment. At the time of this report, she described herself as functionally back to her usual activities and grateful for the close follow-up offered by the multidisciplinary team.

## CRediT authorship contribution statement

**Jeffrey Medina-Medina:** Data curation, Formal analysis, Investigation, Project administration, Visualization, Writing – original draft, Writing – review & editing. **Luis Vargas-González:** Investigation, Visualization, Writing – original draft. **Katia Munguia-Galeano:** Investigation, Visualization, Writing – original draft, Writing – review & editing. **Norwing García-García:** Investigation, Visualization, Writing – original draft, Writing – review & editing. **Melvin Romero-Jarquin:** Investigation, Visualization, Writing – review & editing. **Christian Toval-Ruiz:** Methodology, Visualization, Writing – review & editing. **Manuel López-Urbina:** Investigation, Supervision, Validation, Writing – review & editing.

## Ethics approval and consent to participate

Ethical approval was not required for this case report according to institutional policies. Written informed consent was obtained from the patient for publication of this case report.

## Consent for publication

Written informed consent for publication of the clinical details and accompanying images was obtained from the patient.

## Availability of data and materials

All data generated or analyzed during this study are included in this published article. Additional information is available from the corresponding author upon reasonable request.

## Funding

This research received no specific grant from any funding agency in the public, commercial, or not-for-profit sectors.

## Declaration of competing interest

The authors declare that they have no competing interests.

## Abbreviations

PBML: Pulmonary benign metastasizing leiomyoma; HRCT: High-resolution computed tomography; CT: Computed tomography; FNA: Fine-needle aspiration; H&E: Hematoxylin and eosin; IHC: Immunohistochemistry; SMA: Smooth muscle actin; ER: Estrogen receptor; TTF-1: Thyroid transcription factor-1; ^18^F-FDG PET/CT: ^18^F-fluorodeoxyglucose positron emission tomography/computed tomography; BMI: Body mass index; CARE: CAse REport (guidelines); PEComa: Perivascular epithelioid cell tumor. ACR: American College of Radiology; TI-RADS: Thyroid Imaging, Reporting and Data System.

## References

[1] Ye Z., Wu X., Fang C., Zhu M. (2025). Pulmonary benign metastasizing leiomyoma: a retrospective analysis of seven cases including a rare coexistence with in situ mucinous adenocarcinoma. Biomedicines.

[2] Wojtyś M.E., Kacalska-Janssen O., Ptaszyński K., Lisowski P., Kunc M., Wójcik J. (2022). Benign metastasizing leiomyoma of the lung: diagnostic process and treatment based on three case reports and a review of the literature. Biomedicines.

[3] Fan R., Feng F., Yang H., Xu K., Li S., You Y. (2020). Pulmonary benign metastasizing leiomyomas: a case series of 23 patients at a single facility. BMC Pulm. Med..

[4] Riley D.S., Barber M.S., Kienle G.S., Aronson J.K., von Schoen-Angerer T., Tugwell P. (2017). CARE guidelines for case reports: explanation and elaboration document. J. Clin. Epidemiol..

[5] Patton K.T., Cheng L., Papavero V., Blum M.G., Yeldandi A.V., Adley B.P. (2006). Benign metastasizing leiomyoma: clonality, telomere length and clinicopathologic analysis. Mod. Pathol..

[6] Barnas E., Ksiazek M., Ras R., Skret A., Skret-Magieroo J., Dmoch-Gajzlerska E. (2017). Benign metastasizing leiomyoma: a review of current literature in respect to the time and type of previous gynecological surgery. PLoS One.

[7] Piórek A., Płużański A., Wiśniewski P., Tabor S., Winiarczyk K., Knetki-Wróblewska M. (2024). Pulmonary benign metastasizing Leiomyoma in a postmenopausal woman: a case report and review of the literature. Diseases.

[8] Su H., Fan R., Yang H., You Y., Zhu L., Feng F. (2023). Pulmonary benign metastasizing leiomyoma in patients aged 45 years and younger: clinical features and novelty in treatment. BMC Pulm. Med..

[9] Fasih N., Shanbhogue A.K.P., Macdonald D.B., Fraser-Hill M.A., Papadatos D., Kielar A.Z. (2008). Leiomyomas beyond the uterus: unusual locations, rare manifestations. Radiographics.

[10] Constantinescu A., Stoicescu E.R., Iacob R., Chira C.A., Cocolea D.M., Nicola A.C. (2024). CT-Guided transthoracic core-needle biopsy of pulmonary nodules: current practices, efficacy, and safety considerations. J. Clin. Med..

[11] Nucci M.R., Drapkin R., Dal Cin P., Fletcher C.D.M., Fletcher J.A. (2007). Distinctive cytogenetic profile in benign metastasizing Leiomyoma: pathogenetic implications. Am. J. Surg. Pathol..

